# Bibliographical

**Published:** 1887-11

**Authors:** 


### BIBLIOGRAPHICAL.

THE AMERICAN SYSTEM OF DENTISTRY. In Treatises by various Authors. Edited by WILBUR F. LITCH, M. D., D. D. S. Philadelphia: Lea Brothers & Co. 1887.

This is the most voluminous and pretentious work upon the subject of dentistry that has yet been published in America. It consists of three volumes of more than one thousand pages each, profusely illustrated and beautifully printed. The articles contained cover a wide range of subjects, and the authors were selected as those most competent to write upon the designated themes. In reviewing a work of this magnitude, composed of so many separate papers, it will be impossible, within the limits which this journal affords, to consider each contribution in detail. We must speak of it as a whole.

We think that the title of the work does not definitely express its scope and character. A "system" of dentistry presupposes a regular connection of parts forming an entire whole, which shall give a consistent exposition of all the principles and practice of the science and art of dentistry. Each chapter should be a sequel to its predecessor, and there should be a regular succession of the various themes, all blended into one harmonious whole. The work under notice consists of separate, isolated monographs, written by different authors, without special reference to or knowledge of those to which they were to be joined. As a consequence, there is sometimes a painful lack of harmony in the views expressed, and the theories inculcated are at variance with each other.

The principal fault of the work as a "system," then, is its lack of connection. This defect is inherent to the plan upon which it was projected. When to an author of acknowledged eminence is committed the preparation of an essay upon a designated subject, he must be given entire latitude in the development of his views. The papers cannot be "edited" into harmony with the theories possibly held by others, for each author is responsible for his own teachings. The editor can do little more than collocate the material furnished him. Hence, instead of a congruous procession of themes, each paper is as complete by itself and bears as little relation to the others as do the original articles in a good dental journal. A proper "system" of dentistry will not be produced save by individual effort, or by intimate collaboration of joint authors.

In the work under notice, it often occurs that some of the most important subjects are considered with comparative brevity, while others of less importance to the practicing dentist are continued to interminable lengths. The subject-matter and its import to practitioners seems seldom to have dictated the amount of space which should be devoted to it. As an instance, oral surgery occupies, in two articles by different authors, but eighty pages, while the moulding and carving of porcelain teeth, which is almost peculiar to manufacturers of dental goods, has sixty pages. That important subject, diseases incident to the first dentition, receives twenty-eight pages, while anæsthesia, which properly belongs to the study of general medicine, is spun out to two hundred pages.

The work as a whole, then, is not without defects, As a "system" of dentistry, as a text-book for students, as a work for every-day reference by the practitioner, we cannot think that it will supersede or even take rank with such treatises as Garretson, and Tomes, and Heath, in Surgery; Gorgas, in Therapeutics; Salter, and Wedl, in pathology; Richardson, and Hunter, in Mechanics, or Taft, in Operative Dentistry, although it sometimes contains the results of later investigations.

As a collection of essays and papers upon dental subjects it is unrivaled, and in this light it should be read, and in this view it is well worth the reading. There are single essays which, carefully studied, will repay the purchaser of a volume for the full outlay. They may contain little or nothing that is entirely new, but in the main they are the crystallization of the thoughts and observations of a life-time. Most of the authors have expressed substantially the same ideas at other times and in other places, but here we have the summing up of the whole, a condensation of the most important of the work of the dental journals for the past ten years. While we do not believe that "The American System of Dentistry" is adapted to or will soon take its place as a standard authority or text-book in dental science, it contains matter which should be within the reach of every progressive practitioner, and certainly no dental library will be complete without it.

The publishers are to be complimented upon the clear typography, the neat general appearance, and especially upon the excellence of the illustrations, of which there are nearly *nineteen hundred* in the three very handsome volumes.

Differential Diagnosis; A Manual of the Comparative Semeiology of the More Important Diseases. By F. de Haviland Hall, M. D., Assistant Physician to the Westminster Hospital, London. Third American edition. Thoroughly revised and greatly enlarged. Edited by Frank Woodbury, M. D. Philadelphia: D. G. Brinton. 1887.

We cannot see how a man engaged in general practice can well do without such a work as this. In the treatment of any pathological condition the first essential is a clear diagnosis. Many diseases bear such a close resemblance to each other, especially in the prodromal stages, that their early symptoms are likely to be confounded. It is only by a careful comparison of conditions and indications that the practitioner is enabled to anticipate complications, or even promptly to meet exigencies as they arise. But it is impossible to carry in the mind, without confusion, all the distinguishing characteristics which designate special diseases, while the life of a patient may depend upon an early and correct diagnosis. Hence the necessity for a reliable hand-book of reference for the comparison of symptoms in allied disorders, and in Hall's "Differential Diagnosis" we have them, frequently arranged in parallel columns. We cannot more forcibly recommend the book than by the presentation of a page from it, comparing the symptoms of Typhoid and Typho-Malarial Fevers:

| TYPHOID | TYPHO-MALARIAL |
| --- | --- |
| Occurs in all localities, most common in the North. | Only in miasmatic localities, most common in the South. |
| Invasion gradual and without remittance. | Often begins as simple intermittent or remittent. |
| Daily exacerbation and remission slight. | Daily exacerbation decidedly marked. |
| Diarrhœa the rule Tympanites common. Abdominal tenderness considerable; epigastric and hepatic tenderness slight. | Constipation the rule. Tympanites rare. Abdominal tenderness slight; epigastric and hepatic tenderness considerable. |
| Temperature comparatively low. Delirium low and muttering. | Temperature high, especially at the outset. Delirium active. |
| Spleen not involved to the same extent. | Tumefaction of spleen very marked. |
| Sordes on the teeth the rule. | Sordes rare. |
| Peyer's glands always involved. | Peyer's glands rarely involved. |
| Rose-colored eruption present. | Eruption generally entirely absent. |
| Pigment deposits absent | Pigment deposits in various tissues and organs very common. |

A PRACTICAL TREATISE ON THE DISEASES OF THE HAIR AND SCALP. By GEORGE THOMAS JACKSON, M. D. New York: E. B. Treat. 1887. Price, $2.75.

As medical knowledge increases and physicians become more and more familiar with the minute changes in the tissues, consequent upon diseased conditions, the greater becomes the necessity for works like that under notice, exclusively devoted to the consideration of the pathological changes in a single organ or tissue. A proper study of the symptomatology and treatment of all diseases, even of a series of organs, within the limits of a single volume, is impossible. Who would have thought, a few years ago, that a volume of 350 pages could be profitably devoted to a study of diseases of the hair and scalp? Yet if any one still entertains doubt upon the subject, a brief examination of this one will give convincing evidence.

There is scarce a man who is not vitally interested in the subject-matter of this book. Alopecia, or baldness, for instance, is becoming frightfully common, and few laymen can give an intelligent opinion of even one of its causes. Physicians, too, are lamentably ignorant concerning this so common affection, and few are competent to prescribe remedial or prophylactic measures. A careful study of this volume will prove of lasting benefit to every one, whether professional or belonging to the laity.

A very complete bibliography of the subject is appended, and this will prove of benefit to any one who desires to make a study of the literature of the subject.

INSANITY. ITS CLASSIFICATION, DIAGNOSIS AND TREATMENT. A Manual for Students and Practitioners of Medicine. By E. C. SPITZKA, M. D. New York: E. B. Treat, 1887. Price, $2.75.

Like the book noticed above, from the same press, this is one of the volumes of a series called "Medical Classics," being hand-books upon definite pathological conditions, each the work of an author eminent in his special field. Dr. Spitzka needs no introduction to the members of the medical profession. During the past five years his name has been as frequently upon the lips of the reading public as that of any alienist or neurologist in this country. His views are well known, and he has, perhaps, contributed as much as any one to popular instruction upon insanity, and to the comprehension that it is a symptom of a pathological condition. The poor diseased lunatic is no longer looked upon as one possessed of a devil, or as the victim of his own vices. Instead of chains and scourges, insane asylums now employ remedial and hygienic measures for the relief of the demented.

The work under notice considers insanity according to the usually accepted classification, and describes the symptoms and prescribes the proper treatment. It is illustrated with cuts of diseased conditions of brains, and is enriched with the personal experiences of the author, which have been extensive and varied. There are directions for the examination of the insane, and for the recognition of simulation. Altogether, the book is very complete, and will be indispensable to every neurologist.

LESSONS IN GYNECOLOGY. By WILLIAM GOODELL, A. M., M. D., Professor of Clinical Gynecology in the University of Pennsylvania. Third edition. Revised and greatly enlarged, with one hundred and twelve illustrations. Philadelphia: D. G. Brinton. 1887.

Any book which bears the name of the accomplished Dr. Brinton, whether as author or publisher, is certain to attract the attention of medical men, for there are few who possess such an accurate knowledge of, or such consummate skill in judging, that which will prove both interesting and instructive in literature. This book contains the substance of the clinical and didactic lectures of the author, delivered to the third-year students of the Medical Department of the University of Pennsylvania. The first and second editions were long ago exhausted, and it has for some time been out of print, the author only lately having found time to revise and enlarge it. It does not pretend to be a complete treatise upon all the diseases of women, but takes up the more common gynecological affections and considers them from a clinical standpoint. It is singularly clear and lucid in its pathological descriptions, and the operations and treatment recommended are made so plain that the physician who fails to comprehend must be possessed of singular obtuseness. This distinct intelligibility is one of the principal charms of the work, and makes it invaluable to the young practitioner for study, and to the older one for reference. Too many medical books are written by men who have, perhaps, a sufficiency of knowledge of their subject, but who lack that easy command of language, that ready facility of expression, that happy faculty of dressing a thought in the appropriate words to clearly and forcibly impress it upon the mind, and to bring into prominence the salient points which are so essential to a well-written book. There are many profound authors whom it is almost impossible to follow through a chapter, because of their lack of perspicuity. Such an objection cannot be urged against the author of "Lessons in Gynecology." The student will peruse it with interest, and will derive more advantage from a chapter of his easily comprehended style than from volumes of the labored paragraphs of authors destitute of literary ability.

MASSAGE. The Principles and Practice of Remedial Treatment by Imparted Motion; Mechanical Processes. By GEO. H. TAYLOR, M. D. New York: John B. Alden, Publisher. 1887.

Manual and mechanical manipulation, or passive exercise and communicated motor energy for the treatment of certain diseased conditions, have, of late years, attracted much attention. This work of 173 pages is devoted to a consideration of the principles underlying intelligent massage, and to the manual and mechanical motions most conducive to the end sought. It is plainly written, and is illustrated with cuts, which make the subjects spoken of clear and intelligible.

THE PHYSICIANS' VISITING LIST FOR 1888.

This is the thirty-seventh year of publication of this best of the visiting lists, by Lindsay & Blakiston, and their successors, P. Blakiston, Son & Co. It contains everything which such a work should contain, and much which will not be found in others of the kind. The volume for this year contains improvements over its predecessors, which were thought in their day to be perfect.

THE DENTAL CHAIR. A Poem of Lights and Shadows. By GEO. H. CHANCE, D. D. S., Portland, Oregon.

Practitioners of dentistry are usually too deeply immersed in the practical and prosy details of every-day work to woo the muses with much success. Dr. Chance, however, mounts Pegasus and indulges in a somewhat sustained flight, during which he takes occasion to express his mind very freely upon certain professional and unprofessional matters, and it would redound to the credit of dentistry were some of his criticisms better heeded.

TRANSACTIONS OF THE TEXAS STATE MEDICAL ASSOCIATION FOR 1887.

It takes a volume of 433 pages to record the doings of this society. In some of the older States a much smaller book contains all that is said and done worth recording. But this was prepared by a medical journalist, and that accounts for its completeness. To Dr. F. E. Daniel, Secretary, and the editor and proprietor of *Daniel's Texas Medical Journal,* is the society indebted for such a complete record.

TRANSACTIONS OF THE ILLINOIS STATE DENTAL SOCIETY FOR 1887.

We know of no State Society which annually presents so much of important matter to the profession as does that of Illinois. The present volume is a worthy continuation of a valuable series.

*Report of the Maryland State Board of Health for* 1887. The Sanitation of Cities and Towns and the Agricultural Utilization of Excretal Matters.

*Intubation of the Larynx.* Papers read before the New York Academy of Medicine, at the stated meeting of June 2, 1887. Reprinted from the Medical Record.

*The Radical Cure of Retro-displacements of the Uterus and Procidentia, by Alexander's Operation and Median Corporraphy.* By J. H. KELLOGG, M. D. Reprinted from the Transactions of the Michigan State Medical Society, 1887.

*The Inconsistency of our Code of Dental Ethics.* By Dr. C. H. LAND, Detroit, Mich.

*Recent Advances in Preventive Medicines.* By GEO. H. ROHE, M. D. Reprinted from the Journal of the American Medical Association.

*Some Important Points in the Treatment of Deep Urethral Stricture.* By F. N. OTIS, M. D. Reprinted from the New York Medical Journal.

*Ovarian Tumors and Remarks on Abdominal Surgery.* By EDWARD BORCK, A. M., M. D., St. Louis, Mo.

*A Review of the Most Important Advances in Surgery, Medicine and Pharmacy in the last forty years.* By C. W. MOORE, M. D. Reprinted from the Pacific Record of Medicine and Surgery.

